# The Fungal Pathogen *Moniliophthora perniciosa* Has Genes Similar to Plant PR-1 That Are Highly Expressed during Its Interaction with Cacao

**DOI:** 10.1371/journal.pone.0045929

**Published:** 2012-09-20

**Authors:** Paulo J.P.L. Teixeira, Daniela P.T. Thomazella, Ramon O. Vidal, Paula F.V. do Prado, Osvaldo Reis, Renata M. Baroni, Sulamita F. Franco, Piotr Mieczkowski, Gonçalo A.G. Pereira, Jorge M.C. Mondego

**Affiliations:** 1 Departamento de Genética, Evolução e Bioagentes, Universidade Estadual de Campinas, Campinas, São Paulo, Brazil; 2 Laboratório Nacional de Biociências, Campinas, São Paulo, Brazil; 3 Centro de Pesquisa e Desenvolvimento em Recursos Genéticos Vegetais, Instituto Agronômico de Campinas, Campinas, São Paulo, Brazil; 4 Department of Genetics, School of Medicine, University of North Carolina at Chapel Hill, Chapel Hill, North Carolina, United States of America; University of Sydney, Australia

## Abstract

The widespread SCP/TAPS superfamily (SCP/Tpx-1/Ag5/PR-1/Sc7) has multiple biological functions, including roles in the immune response of plants and animals, development of male reproductive tract in mammals, venom activity in insects and reptiles and host invasion by parasitic worms. Plant Pathogenesis Related 1 (PR-1) proteins belong to this superfamily and have been characterized as markers of induced defense against pathogens. This work presents the characterization of eleven genes homologous to plant *PR-1* genes, designated as *MpPR-1*, which were identified in the genome of *Moniliophthora perniciosa*, a basidiomycete fungus responsible for causing the devastating witches' broom disease in cacao. We describe gene structure, protein alignment and modeling analyses of the MpPR-1 family. Additionally, the expression profiles of *MpPR-1* genes were assessed by qPCR in different stages throughout the fungal life cycle. A specific expression pattern was verified for each member of the *MpPR-1* family in the conditions analyzed. Interestingly, some of them were highly and specifically expressed during the interaction of the fungus with cacao, suggesting a role for the MpPR-1 proteins in the infective process of this pathogen. Hypothetical functions assigned to members of the *MpPR-1* family include neutralization of plant defenses, antimicrobial activity to avoid competitors and fruiting body physiology. This study provides strong evidence on the importance of *PR-1-like* genes for fungal virulence on plants.

## Introduction

The basidiomycete fungus *Moniliophthora perniciosa* is the causative agent of witches' broom disease (WBD) in cacao. This devastating disease is responsible for large losses in cacao plantations in the Americas and is a potential threat to other cacao-growing areas throughout the world [1], [2]. *M. perniciosa* displays a hemibiotrophic lifestyle, with sequential biotrophic (infective) and necrotrophic stages in the plant. These two mycelial stages are morphologically distinct: whereas the biotrophic mycelium is monokaryotic, the necrotrophic stage is dikaryotic and presents clamp connections for nuclei transfer.

The disease cycle initiates when fungal basidiospores infect meristematic tissues of cacao – such as shoots, fruits and floral cushions – where they germinate and develop as biotrophic monokaryotic hyphae. *M. perniciosa* does not use any specialized infection structure to enter the plant (i.e. apressorium), as observed for the majority of biotrophic and hemibiotrophic fungi [3]. This fungus enters the host tissues through stomata or wounds and colonizes the plant apoplast as thick monokaryotic hyphae. In this stage of the disease, the parasitic fungus causes drastic morphophysiological alterations in the host, resulting in the formation of hyperplastic and hypertrophic stems, known as green brooms. During the disease progression, the pathogen switches to its necrotrophic dikaryotic stage, which parallels the death of the infected plant tissue. In this phase of WBD, known as dry broom, *M. perniciosa* colonizes the dead plant and can be found in the intracellular spaces of cacao. After alternating wet and dry periods, the fungus produces basidiomata that release basidiospores, reinitiating the disease cycle [1], [2].

During recent years, efforts have been directed to develop a solution to control this disease. In 2000, the WBD genome initiative (www.lge.ibi.unicamp.br/vassoura) was launched and, since then, it has supported several molecular and biochemical studies involving both the pathogen and the plant [4]–[9]. With the recent technological advances in the area of DNA sequencing, transcriptomes representing a variety of growth and developmental conditions of *M. perniciosa* – including transcriptomes of the fungus developing *in planta* – were sequenced using the RNA-seq technology. As a result, a comprehensive database named WBD Transcriptome Atlas has been constructed, and has contributed important information on the molecular basis of the *M. perniciosa*-cacao interaction (Teixeira *et al*., manuscript in preparation).

The establishment of a disease process depends on the ability of the pathogen to overcome or neutralize plant defenses and then initiate a parasitic relationship with its host. However, to halt pathogenic colonization, plants have developed an arsenal of defense responses, which include induction of pathogenesis-related (PR) genes [10], production of secondary metabolites as well as reinforcement of cell walls. Also, usually triggered by the recognition of a pathogen attack, plants produce highly toxic radicals, such as nitric oxide and reactive oxygen species, which can lead to the establishment of a local cell death (the hypersensitive response, HR). Among the induced pathogenesis-related genes, PR-1s have been frequently identified and used as markers of plant defense responses [10]. Notably, they were shown to have microbicide activity against oomycetes and fungi [11]–[14].

PR-1 proteins are members of a superfamily named SCP/TAPS (Sperm-Coating Protein/Tpx-1/Ag5/PR-1/Sc7) or CAP (Cysteine-rich secretory proteins, Antigen 5, and Pathogenesis-related 1). This superfamily has members throughout the eukaryotic kingdom, suggesting an important role for these proteins in the biology of eukaryotes [15], [16]. Thus far, only a single report has shown the existence of enzymatic activity for a SCP/TAPS protein [17]. The protein Tex31 of the predatory marine mollusk *Conus textile* showed serine-proteolytic activity against a specific pro-peptide precursor of a venom toxin [17]. In addition, structural analyses indicated that four highly conserved amino acids (two histidines and two glutamates) form the putative catalytic site of SCP/TAPS proteins [16]–[22]. Although the existence of biochemical activity has not been shown for any other SCP/TAPS proteins, they are associated with various biological processes, such as male reproductive tract development [23], [24], immune responses in plants and animals [25], venom activity of reptiles and insects [26]–[29] and host invasion by parasites [30]–[32].

In fungal species, SCP/TAPS proteins have been studied in *Saccharomyces cerevisiae*, in which they are highly expressed under nutrient starvation conditions [33]. In the basidiomycete *Schizophyllum commune*, SCP/TAPS proteins have been associated with fruiting body formation [34]. Interestingly, in the ascomycetes *Candida albicans* and *Fusarium oxysporum*, deletion of a SCP/TAPS gene impaired virulence on animals, indicating a role for this class of genes in fungal pathogenicity [35], [36]. Considering that PR-1 proteins are widespread markers of the induced defense response in plants, what would be the function of their homologs in a plant pathogenic fungus, such as *M. perniciosa*?

This article describes the identification of a SCP/TAPS family in the *M. perniciosa* genome, the analysis of structural features of these genes, and their expression profile throughout *M. perniciosa* development. *M. perniciosa* SCP/TAPS proteins were modeled, and some structural differences were revealed among them. Based on these results, we present a hypothetical model in which SCP/TAPS proteins play a role in *M. perniciosa* pathogenicity by interfering with the defense system of cacao plants.

## Results

### Characterization of the *PR-1* gene family in *M. perniciosa*

Annotation of a genome draft of *M. perniciosa* [7], and inspection of fungal EST libraries [6], [8] identified four *PR-1*-like genes in this pathogen (*MpPR-1a* to *MpPR-1d*). Later, improvements in the genome assembly obtained with next generation sequencing data (unpublished data) allowed the identification of seven additional members of the *MpPR-1* family (*MpPR-1e* to *MpPR-1k*), totaling eleven *PR-1*-like genes in *M. perniciosa*. These members are very heterogeneous in size and gene structure, with coding sequences (CDS) ranging from 447 to 1,152 nucleotides and intron composition varying between two to five introns. Three of these genes (*MpPR-1c*, *MpPR-1d* and *MpPR-1j*) are organized *in tandem*. Sequence details of these eleven genes are shown in Table 1, and their respective structures (exon-intron positions) are depicted in figure S1.

**Figure 1:**
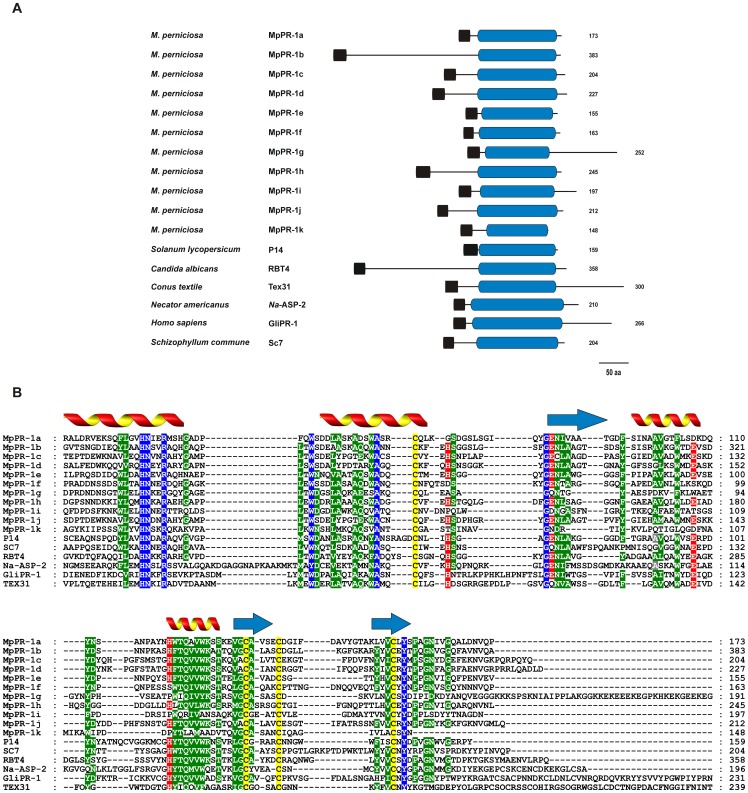
Comparison of MpPR-1 and SCP/TAPS proteins of representative organisms. (A) Domain arrangement of SCP/TAPS proteins. Hydrophobic signal peptides are shown in black and SCP/TAPS domains are represented in blue. The numbers on the right show the size of each protein. Large N-terminal and C-terminal expansions are observed in MpPR-1b and MpPR-1g, respectively. (B) Alignment of the conserved domain of SCP/TAPS proteins. In general, the SCP/TAPS superfamily members show similarities only over the SCP/TAPS domain. Conserved residues (100% of identity) are shown in blue and semi-conserved residues (at least 60% of identity) in green. Putative active site residues are highlighted in red and cysteines in yellow. Secondary structure elements are shown above the alignment (arrow: β-sheets; helix: α-helixes). P14, tomato PR-1 (GenBank P04284); RBT4, repressed by TUP1 from *Candida albicans* (GenBank AAG09789); Tex31, SCP/TAPS from the mollusk *Conus textile* (GenBank CAD36507); Na-ASP-2, *Necator americanus* secreted protein (GenBank AAP41952); GliPR-1, human glioma PR-1 protein (GenBank P48060); SC7, SCP/TAPS from the basidiomycete *Schizophyllum commune* (GenBank P35794).

**Table 1:** Characteristics of the eleven *MpPR-1* genes identified in the *M. perniciosa* genome.

Gene name	GenBank accession number	CDS size (bp)	Number of introns	Protein size (aa)	Signal peptide	BlastX First Hit (Swissprot)*	BlastP First Hit (NCBI-NR)*
*MpPR-1a*	JN620340	522	2	173	Yes (NN score = 0.894)	SC14 (1e-19) *Schizophyllum commune*	XP_001876569.1- predicted protein (1e-54) *Laccaria bicolor*
*MpPR-1b*	JN620341	1152	5	383	Yes (NN score = 0.844)	SC7 (3e-25) *Saccharomyces cerevisiae*	XP_001873270.1*- predicted protein* (2e-63) *Laccaria bicolor*
*MpPR-1c*	JN620342	615	4	204	Yes (NN score = 0.898)	PRY1 (1e-28) *Saccharomyces cerevisiae*	XP_001889714.1- predicted protein (2e-48) *Laccaria bicolor*
*MpPR-1d*	JN620343	684	5	227	Yes (NN score = 0.951)	PRY1 (3e-31) *Saccharomyces cerevisiae*	XP_001889714.1*-* predicted protein (8e-66) *Laccaria bicolor*
*MpPR-1e*	JN620344	468	3	155	Yes (NN score = 0.919)	PR-1C (6e-35) *Nicotiana tabacum*	XP_003038868.1- hypothetical protein (2e-45) *Schizophyllum commune*
*MpPR-1f*	JN620345	492	3	163	Yes (NN score = 0.947)	SC7 (4e-25) *Schizophyllum commune*	CCA68148 – related to PRY1 (8e-42) *Piriformospora indica*
*MpPR-1g*	JN620346	759	4	252	Yes (NN score = 0.905)	SC7 (4e-13) *Schizophyllum commune*	EFY95292.1 – hypothetical protein (4e-15) *Metarhizium anisopliae*
*MpPR-1h*	JN620347	738	5	245	Yes (NN score = 0.804)	PR-1B (5e-24) *Nicotiana tabacum*	XP_001828886.1- hypothetical protein (3e-41) *Coprinopsis cinerea*
*MpPR-1i*	JN620348	498	3	165	Yes (NN score = 0.940)	SC7 (4e-05) *Schizophyllum commune*	EGO02028.1- hypothetical protein (3e-10) *Serpula lacrymans*
*MpPR-1j*	JN620349	639	4	212	Yes (NN score = 0.918)	PRY3 (8e-30) *Saccharomyces cerevisiae*	XP_001889714.1- predicted protein (2e-54) *Laccaria bicolor*
*MpPR-1k*	JN620350	447	2	148	Yes (NN score = 0.944)	P14 (2e-03) *Solanum lycopersicum*	XP_002578075.1- venom allergen 21 (4e-09*) Schistosoma mansoni*

* e-values are shown in parentheses.

**Figure 2:**
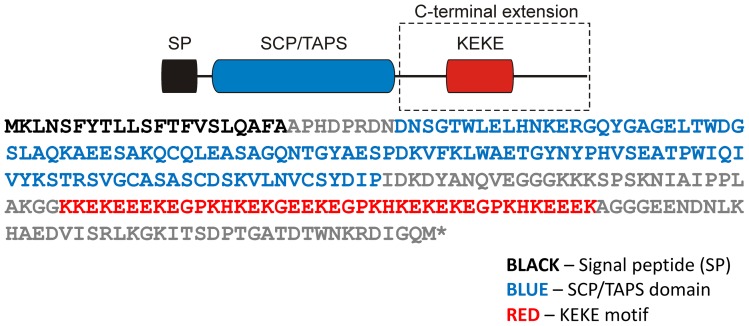
Domains identified in the MpPR-1g protein. In addition to the SCP/TAPS domain, this protein has a KEKE motif in its C-terminal extension. This motif is known to mediate the interaction with other proteins or ions.

Hydrophobic signal peptide sequences predicted with the TargetP program [37] were identified in all eleven *MpPR-1* sequences (NN score >0.80), strongly suggesting that these proteins are secreted. Additionally, all MpPR-1 proteins showed a single SCP/TAPS domain (InterPro ID IPR014044), as predicted by the InterProScan server [38]. These domains were approximately 130 amino acids in length and ranged between 34% and 82% of the total amino acid sequence of an individual MpPR-1 protein (Fig. 1A). In addition to the SCP/TAPS domain, MpPR-1b and MpPR-1g present N-terminal and C-terminal extensions, respectively. No other InterProScan predicted domain was identified in these extensions or in any of the MpPR-1 proteins described. Interestingly, a careful manual inspection revealed that the C-terminal extension of MpPR-1g is rich in residues of lysine (K) and glutamic acid (E) that are mostly organized in alternating positions, resulting in the formation of a “KEKE” motif [39] (Fig. 2). The MpPR-1b N-terminal extension is also a low complexity region, being rich in serine, threonine and proline residues. However, no described motif could be recognized.

Alignment of the amino acid sequences encoded by all *MpPR-1* genes revealed significant similarity only over the SCP/TAPS domains (Fig. 1B). The amino acids proposed to form the putative catalytic site of SCP/TAPS proteins (two histidines and two glutamic acids, shown in red) were identified in six MpPR-1s (MpPR-1b, MpPR-1c, MpPR-1d, MpPR-1e, MpPR-1h and MpPR-1j). In contrast, MpPR-1a presented two, MpPR-1f had only one and MpPR-1g, MpPR-1i and MpPR-1k had none of the four conserved residues (Fig. 1B).

### Protein structure modeling

**Figure 3:**
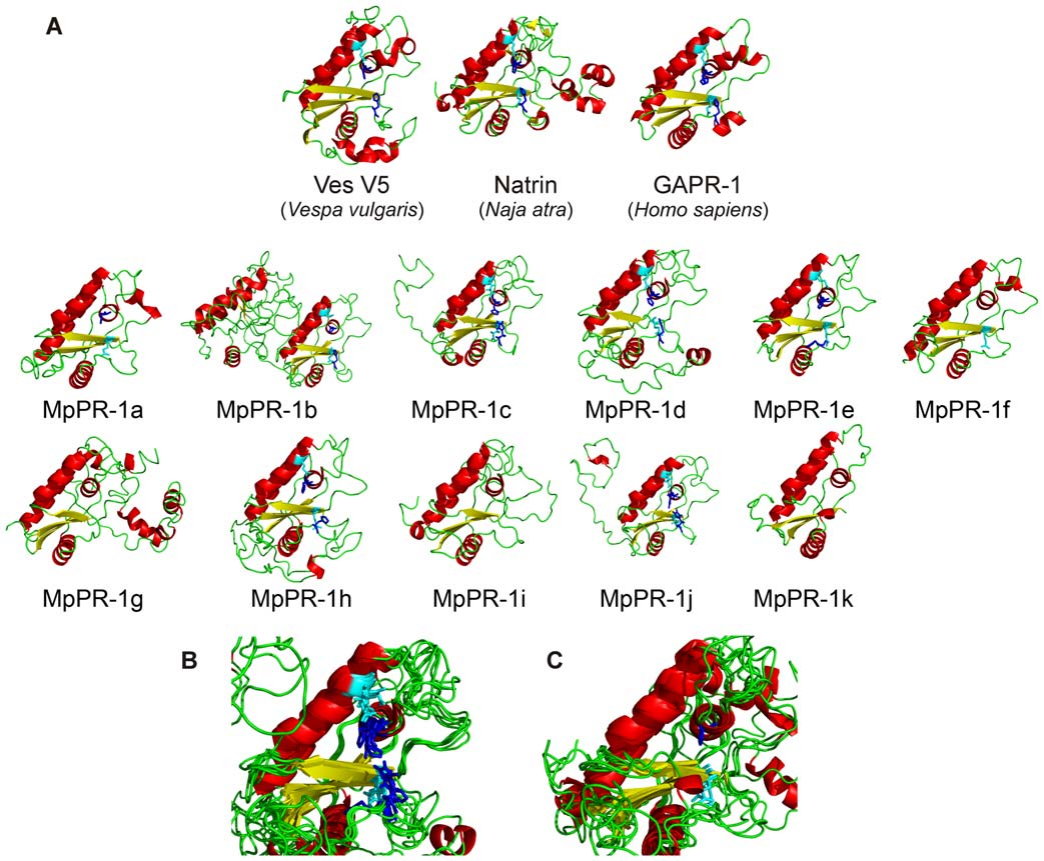
Homology modeling of MpPR-1 proteins. (A) Ribbon stick representation showing the folding of eleven MpPR-1 proteins and three SCP/TAPS proteins used to obtain these models. The putative residues forming the catalytic site are highlighted in dark blue (histidines) and light blue (glutamic acids). Note the presence of an additional protein module in MpPR-1b and MpPR-1g. These modules respectively correspond to the N-terminal and C-terminal extensions observed in these proteins. (B) MpPR-1b, MpPR-1c, MpPR-1d, MpPR-1e, MpPR-1h and MpPR-1j have the four putative active site residues of the SCP/TAPS domain. (C) These residues are partially or completely absent in MpPR-1a, MpPR-1f, MpPR-1g, MpPR-1i and MpPR-1k.

To explore the tertiary structural characteristics within the SCP/TAPS domains of the MpPR-1 family members, we created homology models using the fold prediction metaserver I-TASSER (Fig. 3). The derived models indicated that the MpPR-1 SCP/TAPS domains adopt the α-β-α sandwich conformation, which is common to all superfamily members across the species studied [16], [18] (Fig. 3A). Furthermore, all MpPR-1 proteins possess the large cleft proposed to constitute the SCP/TAPS active site. As mentioned above, six MpPR-1 proteins (MpPR-1b, MpPR-1c, MpPR-1d, MpPR-1e, MpPR-1h and MpPR-1j) have the four conserved residues of the putative catalytic site. These residues are localized within this cleft (Fig. 3B) and are found in the same direction of the orthologous amino acids identified in previous SCP/TAPS crystal structures [18], [21], [22], [25]. These residues are lacking (MpPR-1g, MpPR-1i and MpPR-1k) or partially absent (MpPR-1a and MpPR-1f) in other members of the MpPR-1 family (Fig. 3C), indicating some diversification in their mode of action.

Remarkably, MpPR-1b and MpPR-1g contain two modules: the SCP/TAPS domain and either N-terminal (MpPR-1b) or C-terminal (MpPR-1g) extensions. Protein models indicate that such extensions are structurally organized and have α-helix conformations (Fig. 3A). These additional regions possibly confer a different activity or regulation to these proteins.

### Expression profile of *MpPR-1* genes throughout *M. perniciosa* development

**Figure 4:**
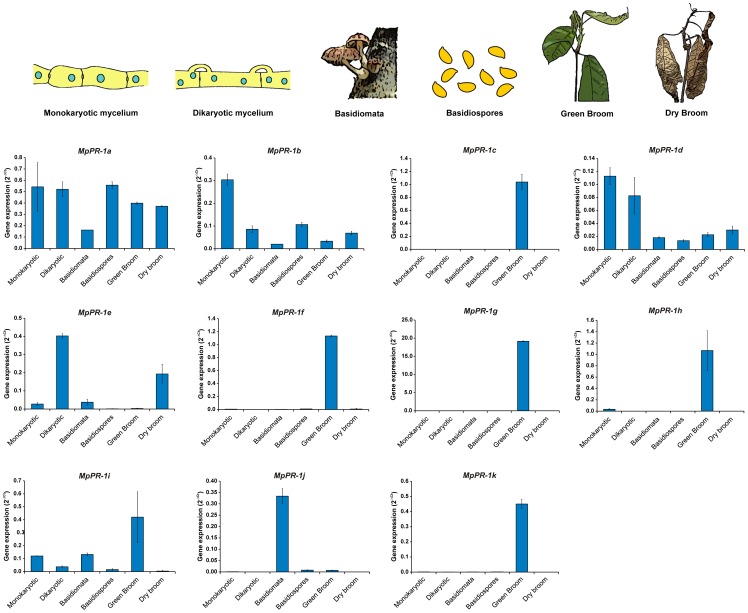
Transcriptional profile of *MpPR-1* family members throughout the *M. perniciosa* life cycle. Each *MpPR-1* gene has a distinct expression profile during fungal development. “Monokaryotic” and “Dikaryotic” hyphae represent the two mycelial stages (biotrophic and necrotrophic) grown under *in vitro* conditions. “Green broom” and “dry broom” correspond to the biotrophic and necrotrophic stages of *M. perniciosa*, respectively, during its interaction with cacao. Analyses were performed by qPCR and the *M. perniciosa β-actin* gene was used as endogenous control to normalize data. Error bars represent standard deviations determined with two biological replicates. Representative drawings of the conditions analyzed are shown on the top.

The expression pattern of *MpPR-1* family members was assessed by quantitative real time PCR (qPCR) throughout the different developmental stages of *M. perniciosa*. As shown in Figure 4, each *MpPR-1* gene showed a specific expression profile: expression of *MpPR-1a*, *MpPR-1b*, *MpPR-1d* and *MpPR-1i* genes was relatively uniform, with nearly similar expression values for most conditions analyzed. In contrast, *MpPR-1e* was up-regulated in the dikaryotic hyphae, whereas *MpPR-1j* was exclusively expressed in mushrooms (basidiomata). Strikingly, five *MpPR-1* genes (*MpPR-1c*, *MpPR-1f*, *MpPR-1g*, *MpPR-1h*, and *MpPR-1k*) were highly expressed during the biotrophic interaction of the fungus with the cacao plant (green broom stage of WBD). These genes were poorly expressed in the necrotrophic hyphae (dry broom stage of WBD) and in the *ex planta* conditions, suggesting a specific role for the encoded proteins in fungal pathogenicity.

### Characterization of an *MpPR-1* cluster

**Figure 5:**
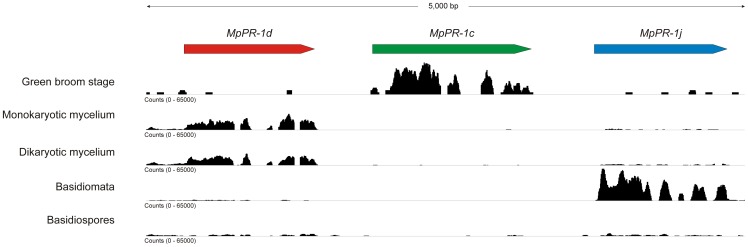
Genomic organization and transcriptional profile of the *MpPR-1* gene cluster found in *M. perniciosa*. The *MpPR-1c*, *MpPR-1d* and *MpPR-1j* genes are arranged *in tandem* over a region of approximately 5 kbp. Analysis of the WBD RNA-seq Atlas shows the expression profile of these *MpPR-1* genes in different conditions (green broom – *in planta* development of the biotrophic monokaryotic hyphae; monokaryotic mycelium; dikaryotic mycelium; basidiomata and basidiospores). Data were visualized using the Integrative Genomics Viewer [62]. The black coverage plot shows cumulative RNA-seq read coverage along the transcripts in all different conditions. Note that these genes were named according to the order they were identified in the fungal genome, and the nomenclature does not necessarily reflect their relative localization in the genome.

As mentioned above, genes *MpPR-1c*, *MpPR-1d* and *MpPR-1j* are arranged *in tandem* over a region of approximately 5 kbp in the *M. perniciosa* genome (Fig. 5). This gene cluster points to the occurrence of gene duplication events during the evolution of the *MpPR-1* family. Indeed, the proteins encoded by these three genes are more similar to each other than to other MpPR-1 members (data not shown). In accordance, these three genes have very similar structures, with a minor difference in *MpPR-1d*, which has an additional intron and a mini-exon following exon 2 (Fig. S1). Importantly, genes *MpPR-1c* and *MpPR-1j* are also highly similar at the nucleotide level (84% identity), indicating a recent event of gene duplication. Despite their similarity, these genes have distinct expression profiles: whereas *MpPR-1j* is highly expressed in basidiomata, *MpPR-1c* is mainly expressed during cacao infection (green broom stage of WBD) (Figs. 4 and 5).

## Discussion

In this study, we identified a family of genes encoding proteins of the SCP/TAPS superfamily in the plant pathogen *Moniliophthora perniciosa*. SCP/TAPS proteins are found in a vast number of organisms, including plants, insects, mammals, fungi, mollusks and worms. Plant Pathogenesis-related proteins (PR-1) belong to this superfamily and are known to accumulate after pathogen invasion [10]. Despite being broadly spread, evidence for the importance of fungal SCP/TAPSs in plant-pathogen interactions has not yet been described.

*M. perniciosa* has a larger number (11) of *SCP/TAPS* genes encoding PR-1-like secreted proteins than other fungal species analyzed to date (Table S1). Although these proteins have a single SCP/TAPS domain, they are very divergent in sequence (Fig. 1B). Some of them show extensions in their N-terminal (MpPR-1b) or C-terminal (MpPR-1g) regions (Fig. 1A and Fig. 3A). Also, six MpPR1s (MpPR-1b, MpPR-1c, MpPR-1d, MpPR-1e, MpPR-1h and MpPR-1j) have all four conserved amino acids (two histidines and two glutamates) of the active site proposed for SCP/TAPS proteins, whereas the other five proteins (MpPR-1a, MpPR-1f, MpPR-1g, MpPR-1i and MpPR-1k) lack the catalytic tetrad (Fig. 1B and Fig. 3). However, the basic signatures of SCP/TAPS domains remain conserved.

The high number of genes and the variation in protein sequence of MpPR-1 members may reflect a process of diversification of this gene family in *M. perniciosa*. In accordance, the cluster including three *MpPR-1* members (Fig. 5) indicates the occurrence of recent events of gene duplication, which are central for the generation of genetic variability. In this regard, functional diversity may occur within this family, and different MpPR-1s likely play a particular role in *M. perniciosa* biology.

Although widely distributed among several species, the functions of SCP/TAPS proteins are still uncertain. Their broad distribution indicates that they play a role in a plethora of biological processes. In the mushroom-forming basidiomycete *Schizophyllum commune*, SCP/TAPS proteins were identified in the basidiomata, being involved in the formation of pseudo-parenchymous tissue of this reproductive structure [34]. In a similar way, *MpPR-1j* is exclusively expressed in the basidiomata of *M. perniciosa*, suggesting a role for this isoform in the physiology/metabolism of fruiting bodies. In contrast, we also identified *SCP/TAPS* genes in the genomes of some basidiomycetes that do not produce mushrooms (e.g., *Ustilago maydis, Puccinia graminis, Melampsora spp* and *Cryptococcus spp*) (data not shown). Therefore, it is likely that SCP/TAPS proteins have functions in basidiomycetes other than fruiting body development/physiology.

In response to pathogen invasion, plants typically produce PR-1 proteins, which have antimicrobial activity and, consequently, are able to inhibit the development of fungi and oomycetes [11]–[13]. Considering that, we hypothesize that some MpPR-1s could play a role in limiting the growth of other microbial competitors (e.g., oomycetes from the genus *Phytophthora*, responsible for causing black pod rot in cacao), thus favoring *M. perniciosa* colonization during WBD progression. Functional experiments are needed to confirm the existence of antimicrobial activity in any of the MpPR-1 proteins identified.

Gene expression analyses revealed that five *MpPR-1* genes (*MpPR-1c*, *MpPR-1f*, *MpPR-1g*, *MpPR-1h* and *MpPR-1k*) are strikingly up-regulated in the green broom stage of WBD, when the fungus grows biotrophically within the plant tissues (Fig. 4). Remarkably, inspection of the WBD RNA-seq Transcriptome Atlas (Teixeira *et al.*, manuscript in preparation) revealed that *MpPR-1g* and *MpPR-1h* are among the most highly expressed genes of *M. perniciosa* during its biotrophic interaction with cacao. Moreover, in addition to the green broom stage, *MpPR-1f* and *MpPR-1h* are notably expressed in germinating basidiospores (Fig. S2), a critical stage for the establishment of infection. None of these genes were significantly expressed in non-germinating spores or in the dry broom stage of WBD (Fig. 4 and Fig. S2), strongly indicating a major role for the encoded proteins in the infective (biotrophic) stage of *M. perniciosa*. Similarly, *SCP/TAPS* genes identified in some animal parasitic worms (*Schistosoma mansoni*, *Brugia malayi*, *Necator americanus* and *Ancylostoma caninum*) are highly expressed in the infective stage and are considered important pathogenicity factors [30], [31], [40]–[42]. In these parasites, SCP/TAPSs are supposed to contribute to their virulence by modulating the host immune response [15], [16].

Recently, a SCP/TAPS protein in the plant-parasitic nematode *Globodera rostochiensis* (Gr-VAP1) was shown to function as an effector by interacting with the tomato cysteine protease Rcr3, which is also a target of the Avr2 effector from the fungus *Cladosporium fulvum* [43]. In addition, SCP/TAPS proteins have been identified in other plant infecting nematodes (e.g. *Heterodera glycines, Meloidogyne incognita* and *Bursaphelenchus xylophilus*), and these are thought to be required for the establishment of parasitism [44]–[48]. Considering the expression pattern of some *SCP/TAPS* genes in *M. perniciosa* and the functions ascribed for the encoded proteins in other pathogenic organisms, it is plausible that some MpPR-1s play a role in the *M. perniciosa*-cacao interaction and may be candidate effectors of this fungal pathogen. Accordingly, a recent study that aimed at the identification of putative effectors in *Melampsora larici-populina* and *Puccinia graminis* reported the enrichment of SCP/TAPSs in the predicted secretome of these rust fungi [49]. A fungal *SCP/TAPS* gene was also identified in EST libraries produced from rye infected with the ascomycete pathogen *Claviceps purpurea* [50], suggesting that these genes might also be important in other plant-fungus interactions.

In recent years, a role for SCP/TAPSs as virulence factors has emerged in many organisms. It is likely that these proteins converged as pathogenicity mechanisms in distinct pathogens/parasites from either plants or animals. Whereas the function of fungal SCP/TAPSs as virulence factors in plant pathogens remains to be confirmed, previous studies demonstrated that these proteins are required for fungal virulence on animals (e.g. *C. albicans* and *F. oxysporum*) [35], [36]. The ascomycete *F. oxysporum* is a multi-host pathogen that is able to infect both plants and animals. Previous work by Prados-Rosales *et al.* verified that *fpr1*, one of the six *SCP/TAPS* genes from this pathogen, is required for fungal virulence on animals but not on plants [36]. Given that the *F. oxysporum* genome contains five other *SCP/TAPS* genes, the absence of a phenotype on plants can be explained by the occurrence of functional redundancy in this gene family. Notably, there is evidence that *fpr1* is part of a gene family that has expanded in *F. oxysporum* and in other plant pathogenic Sordariomycetes [36].

Although the precise activity of SCP/TAPSs is currently unknown, Prados-Rosales *et al.* presented the first genetic evidence for a biological function of the proposed active site of SCP/TAPS proteins [36]. The authors demonstrated that the integrity of the active site is required for *F. oxysporum* virulence on animals. In *M. perniciosa*, six MpPR-1s (MpPR-1b, MpPR-1c, MpPR-1d, MpPR-1e, MpPR-1h and MpPR-1j) contain all four amino acids of the proposed active site. In contrast, the other five proteins do not have the complete catalytic tetrad (Fig. 1B). In this regard, it is possible that *M. perniciosa* PR-1s have distinct mode of actions. For instance, whereas those proteins with the complete catalytic tetrad can function as enzymes, the other PR-1s may act as inhibitors.

Concomitantly to the up-regulation of some *MpPR-1* genes *in planta*, we identified a cacao *PR-1* gene over-expressed in the green broom stage of WBD (Fig. S3). Based on these findings, we suggest that some MpPR-1s could act as competitive inhibitors of the plant PR-1, modulating the cacao immune response. It has already been shown that the SCP/TAPS protein *Na*-ASP-2 of the hookworm *Necator americanus* has a high structural similarity to chemokines, and this protein is proposed to be an antagonistic ligand of receptors that activate the immune system of the vertebrate host [51]. Furthermore, NIF (Neutrophil inhibitory factor), a SCP/TAPS protein from *Ancylostoma caninum*, interferes with the host immune system by interacting with neutrophil receptors [32]. Confirmation of this interesting mechanism in the *M. perniciosa*-cacao interaction may be of primary relevance to the understanding of many other plant diseases and will shed light on our understanding of PR-1 functions.

Among the *MpPR-1* genes that are highly expressed *in planta*, *MpPR-1g* is the only one with a C-terminal extension in addition to the SCP/TAPS domain (Fig. 1A and Fig. 3A). This additional region is rich in lysine (K) and glutamic acid (E) residues, which are mostly organized in alternating positions, resulting in the formation of a "KEKE" motif [39] (Fig. 2). This motif is known to mediate protein-protein associations [39], [52] and is also able to bind divalent ions, such as calcium and zinc [53]. Calcium is an important regulator of many cellular processes, including plant defense responses [54]. In this regard, this additional module may be important in determining the mode of action of MpPR-1g. Whether this protein interacts with other proteins, particularly cacao proteins, and/or interferes with the plant calcium signaling during infection should be the object of future studies.

Overall, this study presents important evidence on the role of fungal SCP/TAPSs in the context of a plant-pathogen interaction. Although the precise function of each MpPR-1 family member is currently unknown, the information provided in our study suggests they have potential roles in some important biological processes, such as fruiting body metabolism, spore penetration and modulation of the host defense response. As a consequence, our results may inform the study of the role of PR-1-encoding genes in other organisms, particularly phytopathogens. Further studies concerning the *M. perniciosa PR-1* gene family will focus on the characterization of this interesting family in terms of fungal development and roles in the *M. perniciosa* interaction with cacao.

## Materials and Methods

### Biological material

Isolate CP02 of *Moniliophthora perniciosa* (Stahel) Aime & Philliphs-Mora [55], was used to perform the experiments. Under *in vitro* conditions, the fungus can only be maintained as a dikaryotic mycelium, and all other developmental stages (basidiomata, basidiospores and monokaryotic mycelium) are obtained from the dikaryotic stage. The reproductive structures (basidiomata) were produced in laboratory according to the protocol described by Pires *et al*. [8]. Fresh basidiomata were used to collect basidiospores according to Frias *et al*. [56].

Basidiospores suspensions were utilized for the *in vitro* production of the monokaryotic mycelia. For this purpose, approximately 3.75×10^5^ basidiospores were inoculated in 125 ml Erlenmeyer flasks containing 50 ml liquid medium (LMCpL+), as described by Meinhardt *et al.* [57]. Liquid cultures were maintained at 28°C and incubated under agitation at 120 rpm. Dikaryotic mycelium was inoculated in the same medium and maintained under the same conditions. Both mycelia were collected 7 days post inoculation to perform the experiments.

*Theobroma cacao L.* cv. “Comum” was used to perform the infection experiments. Three-months-old plantlets were inoculated with 30 µL of a basidiospore suspension (1×10^5^ spores mL^−1^) according to the procedure described by Frias *et al.* [56]. Plantlets were kept in a greenhouse under controlled conditions of temperature (26°C) and humidity (>80%). Green brooms (biotrophic stage) and dry brooms (necrotrophic stage) were collected 30 and 105 days post inoculation, respectively.

### Sequence analysis

Inspection of the *M. perniciosa* genome led to the identification of eleven genes encoding proteins similar to plant pathogenesis-related proteins 1 (PR-1). These genes were named *MpPR-1a* to *MpPR-1k* according to the order they were discovered. The complete open reading frames (ORFs) of these genes were predicted using the program Augustus [58] and confirmed by cDNA sequencing. These sequences have been submitted to GenBank with the accession numbers JN620340 to JN620350. Blast searches were performed using the NCBI-NR and Swissprot databases. Domain prediction of the encoded proteins was performed using the InterProScan server [38] and the presence of a signal peptide for secretion was predicted using the software TargetP 1.1 [37].

### Total RNA extraction and cDNA synthesis

With the exception of basidiospores, samples were ground to a fine powder in liquid nitrogen using a pestle and mortar. Basidiospores walls were broken by vortexing the sample in RNA extraction buffer (Buffer RLT, RNeasy Plant Mini Kit) and 200 mg glass beads (0.4–0.6 µm, Sigma-Aldrich, St. Louis, MO, EUA). RNA isolation was performed using the RNeasy Plant Mini Kit (Qiagen, Valencia, CA, USA) according to the manufacturer's instructions. RNA was treated with DNAse I AmpGrade (Invitrogen, Carlsbad, CA, USA) and its concentration was accessed using the ND-1000 spectrophotometer (NanoDrop, Wilmington, DE, USA). cDNA was synthesized from 1 µg total RNA using the SuperScript II Reverse Transcriptase (Invitrogen), according to the manufacturer's instructions.

### Gene expression assays

Quantitative real time PCR (qPCR) was performed on a StepOne Plus Real Time PCR System (Applied Biosystems, Foster City, CA, USA) using Sybr Green I dye for the detection of PCR products. Each reaction contained 8 μl SYBR Green PCR Master Mix (Applied Biosystems), 250 nM each primer and 50 ng cDNA template in a final volume of 16 μl. No-template reactions were included as negative controls for each set of primers used. The thermal cycling conditions were 94°C for 10 min, followed by 40 cycles of 94°C for 15 s, 53°C for 30 s and 60°C for 1 min, with fluorescence detection at the end of each cycle. In addition, a melting curve analysis was performed to verify the amplification of a single product per reaction. All reactions were conducted in technical triplicates using two independent biological replicates of each sample. The *M. perniciosa β-actin* gene was used to normalize data and expression levels are presented as 2^−ΔCt^. Primers used in this assay are shown in Table S2.

### Protein structure modeling

The fold recognition-based method was implemented using the I-TASSER server [59], which constructed structure models for each MpPR-1 protein using folds of the most similar proteins deposited in the PDB (Protein Data Bank) database (http://www.rcsb.org/pdb). The main templates were based on the structure of three proteins: i) Natrin (PDB – 1xta), a component of the venom of the snake *Naja atras*; ii) GAPR-1 (PDB – 1smb), a SCP/TAPS protein associated with the membrane of the human Golgi system; and iii) Ves V5 (PDB – 1qnx), present in the venom of the wasp *Vespula vulgaris*. The modeled structures were validated by analyzing the Ramachandran plots generated by PROCHECK [60], and the models were displayed using the software PyMOL [61].

## Supporting Information

Figure S1 **Structure of the** ***MpPR-1*** **genes.** Exons are represented by boxes, while introns are shown as lines. Exons are colored to highlight the regions encoding important protein features: predicted signal peptides (black), SCP/TAPS domain (blue) and the remaining ORF (gray).
(TIF)

Figure S2 **Expression levels of** ***MpPR-1*** **genes in germinating and non-germinating basidiospores.** *MpPR-1f* and *MpPR-1h* are highly expressed in germinating basidiospores, supporting a role for the encoded proteins in the establishment of witches' broom disease. Data are part of the WBD Transcriptome Atlas and were obtained by RNA-seq sequencing. Gene expression values are given in Reads Per Kilobase of exon model per Million mapped reads (RPKM).
(TIF)

Figure S3 **Gene expression levels of a cacao** ***PR-1*** **(ID CGD0027635) in infected and healthy plants.** Similar to some *MpPR-1* genes, a cacao *PR-1* (*TcPR-1*) is up-regulated in the green broom stage of WBD. The analysis was performed by qPCR and the *T. cacao α-tubulin* gene (ID CGD0029727) was used as endogenous control to normalize data. Gene IDs refer to the Cacao Genome Database (http://www.cacaogenomedb.org). The qPCR assay was conducted as described in the Material and Methods section and primers used in the experiment were: TcPR-1_F: 5′ ACCTTATGGCGAGAACCTTG 3′, TcPR-1_R: 5′ GGAGTAATCATAGTCGGCCTTC 3′, TcTub_F: 5′ ACCAATCTTAACCGCCTTGTCT 3′ and TcTub_R: 5′ GTTAGTCTGGAACTCAGTCACAT 3′.
(TIF)

Table S1 **Number of** ***SCP/TAPS*** **genes in fungal species with different lifestyles.** Numbers correspond to the genes coding proteins with the InterPro ID IPR014044.
(DOC)

Table S2 **Primers used for quantitative real time PCR analyses of** ***M. perniciosa*** **PR-1 genes.**
(DOC)
