# Supplementary figures and images for: The Fungal Pathogen Moniliophthora perniciosa Has Genes Similar to Plant PR-1 That Are Highly Expressed during Its Interaction with Cacao
Source: PLoS One. 2012 Sep 20;7(9):e45929. doi: 10.1371/journal.pone.0045929 (PMC3447762; doi:10.1371/journal.pone.0045929)

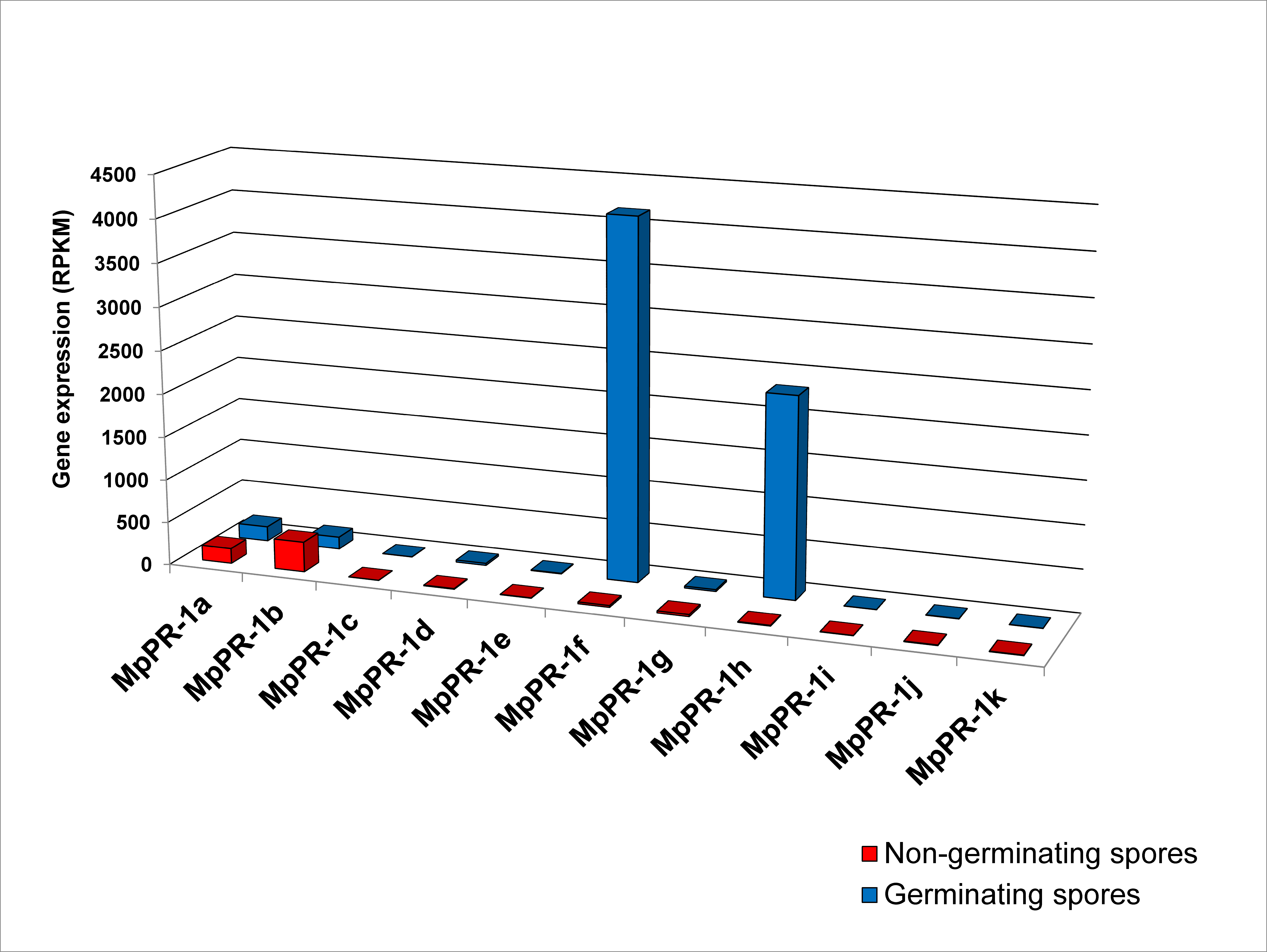

Supplement: Figure S2 — Expression levels of MpPR-1 genes in germinating and non-germinating basidiospores. MpPR-1f and MpPR-1h are highly expressed in germinating basidiospores, supporting a role for the encoded proteins in the establishment of witches' broom disease. Data are part of the WBD Transcriptome Atlas and were obtained by RNA-seq sequencing. Gene expression values are given in Reads Per Kilobase of exon model per Million mapped reads (RPKM). (TIF) [file pone.0045929.s002.tif]

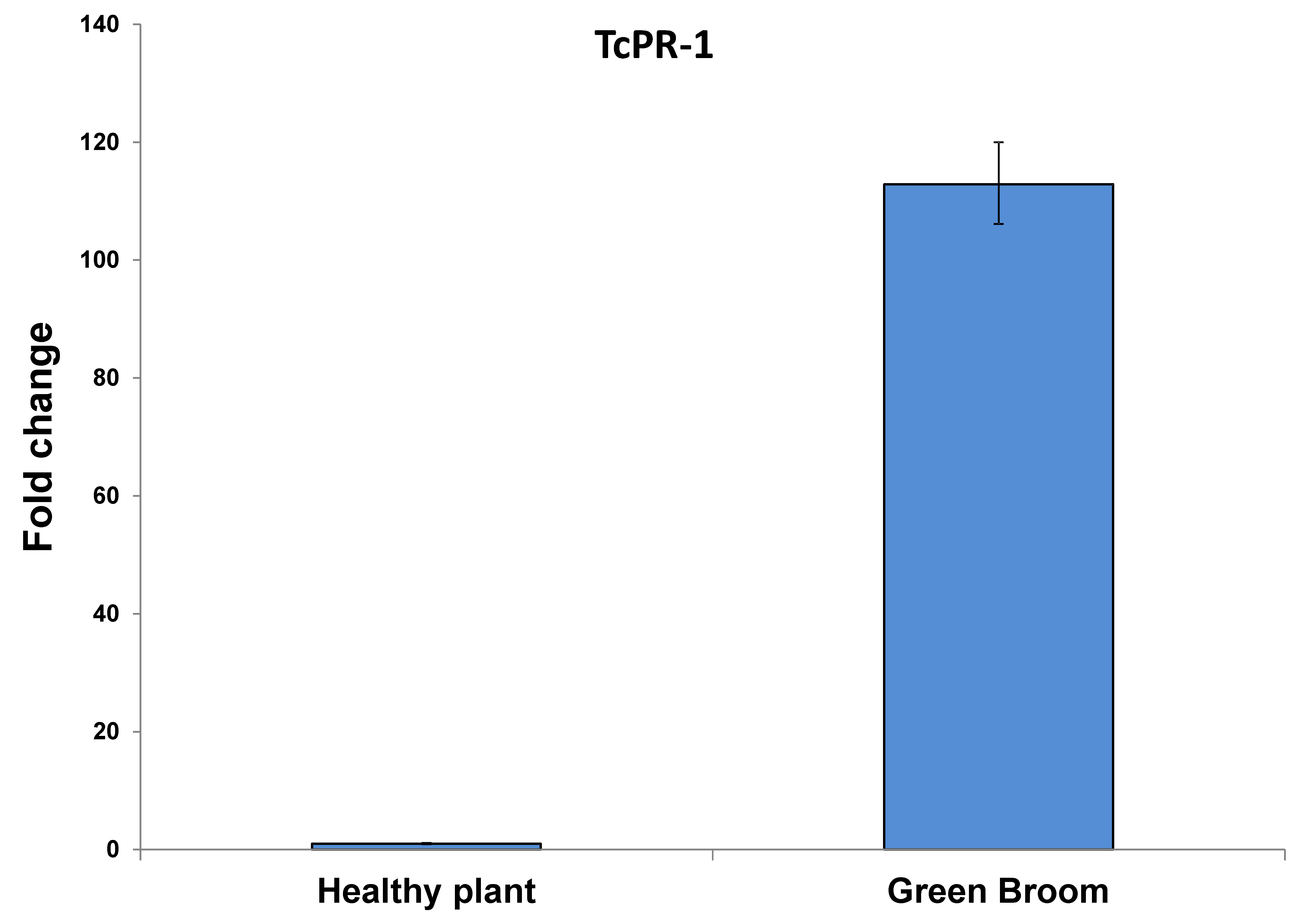

Supplement: Figure S3 — Gene expression levels of a cacao PR-1 (ID CGD0027635) in infected and healthy plants. Similar to some MpPR-1 genes, a cacao PR-1 (TcPR-1) is up-regulated in the green broom stage of WBD. The analysis was performed by qPCR and the T. cacao α-tubulin gene (ID CGD0029727) was used as endogenous control to normalize data. Gene IDs refer to the Cacao Genome Database (http://www.cacaogenomedb.org). The qPCR assay was conducted as described in the Material and Methods section and primers used in the experiment were: TcPR-1_F: 5′ ACCTTATGGCGAGAACCTTG 3′, TcPR-1_R: 5′ GGAGTAATCATAGTCGGCCTTC 3′, TcTub_F: 5′ ACCAATCTTAACCGCCTTGTCT 3′ and TcTub_R: 5′ GTTAGTCTGGAACTCAGTCACAT 3′. (TIF) [file pone.0045929.s003.tif]
