# Supplementary material for: The Fungal Pathogen Moniliophthora perniciosa Has Genes Similar to Plant PR-1 That Are Highly Expressed during Its Interaction with Cacao
Source: PLoS One. 2012 Sep 20;7(9):e45929. doi: 10.1371/journal.pone.0045929 (PMC3447762; doi:10.1371/journal.pone.0045929)
Supplement: Table S1 — Number of SCP/TAPS genes in fungal species with different lifestyles. Numbers correspond to the genes coding proteins with the InterPro ID IPR014044. (DOC) [file pone.0045929.s004.doc]

Supplementary Table 1. Number of *SCP/TAPS* genes in fungal species with different lifestyles. Numbers correspond to the genes coding proteins with the InterPro ID IPR014044.

| **Fungal species** | **SCP/TAPS genes** | **Lifestyle** |
| --- | --- | --- |
| *Botrytis cinerea*^1^ | 5 | Necrotrophic pathogen (Ascomycete) |
| *Sclerotinia sclerotiorum*^1^ | 3 | Necrotrophic pathogen (Ascomycete) |
| *Fusarium graminearum*^1^ | 5 | Hemibiotrophic pathogen (Ascomycete) |
| *Magnaporthe oryzae*^1^ | 7 | Hemibiotrophic pathogen (Ascomycete) |
| *Ustilago maydis*^1^ | 2 | Biotrophic pathogen (Basidiomycete) |
| *Melampsora larici-populina*^2^ | 10 | Biotrophic pathogen (Basidiomycete) |
| *Puccinia graminis*^1^ | 7 | Biotrophic pathogen (Basidiomycete) |
| *Phanerochaete chrysosporium*^2^ | 2 | Saprotrophic (Basidiomycete) |
| *Postia placenta*^2^ | 1 | Saprotrophic (Basidiomycete) |
| *Schizophyllum commune*^2^ | 5 | Saprotrophic (Basidiomycete) |
| *Serpula lacrymans*^2^ | 2 | Saprotrophic (Basidiomycete) |
| *Coprinopsis cinerea*^1^ | 2 | Saprotrophic (Basidiomycete) |
| *Laccaria bicolor*^2^ | 9 | Ectomycorrhizal (Basidiomycete) |
| *Moniliophthora perniciosa* | 11 | Hemibiotrophic pathogen (Basidiomycete) |

^1^ Data from genomes sequenced by the Broad Institute of Harvard and MIT (http://www.broadinstitute.org/)

^2^ Data from genomes sequenced by the Department of Energy Joint Genome Institute ([**http://www.jgi.doe.gov/**](http://www.jgi.doe.gov/))
