# Supplementary material for: The Fungal Pathogen Moniliophthora perniciosa Has Genes Similar to Plant PR-1 That Are Highly Expressed during Its Interaction with Cacao
Source: PLoS One. 2012 Sep 20;7(9):e45929. doi: 10.1371/journal.pone.0045929 (PMC3447762; doi:10.1371/journal.pone.0045929)
Supplement: Table S2 — Primers used for quantitative real time PCR analyses of M. perniciosa PR-1 genes. (DOC) [file pone.0045929.s005.doc]

Supplementary Table 2. Primers used for quantitative real time PCR analyses of *M. perniciosa* *PR-1* genes.

| **Gene name** | **Forward primer** | **Reverse primer** |
| --- | --- | --- |
| *β-actin* | 5’ CCCTTCTATCGTCGGTCGT 3’ | 5` AGGATACCACGCTTGGATTG 3’ |
| *MpPR-1a* | 5` AGTTGAAAGGCTCAGATGGAT 3` | 5` AGTGATTATAGGCGGGATTGG 3` |
| *MpPR-1b* | 5` TAGCGACAGTTATTCCATTTCC 3` | 5` GACTTGAGTTGTGGCTTTCC 3` |
| *MpPR-1c* | 5` CAAACACACAGAACCGACT 3` | 5` GTGGGTTACTGTGTTCATACA 3` |
| *MpPR-1d* | 5` CACTCAAGTTGTCTGGAAGAG 3` | 5` CTGGTAACTTTGCTGGACG 3` |
| *MpPR-1e* | 5’ CCGAGCCTTTGACCTGGA 3’ | 5’ CCCAAGTTCTGTAGTGGATTTCC 3’ |
| *MpPR-1f* | 5’ ACCAGTGCAACTTCCAGACTTC 3’ | 5’ CACACGATTTGGGTCCAGC 3’ |
| *MpPR-1g* | 5’ CTAAGCAATGTCAACTCGAGGC 3’ | 5’ CCCAACACTTCTGGTTGACTTG 3’ |
| *MpPR-1h* | 5’ CCGCGGTTCAGCTATGGC 3’ | 5’ CACGTGGAGCGTAGTGGG 3’ |
| *MpPR-1i* | 5’ CGCAGCTTTGGGGCGATA 3’ | 5’ GGTAGCTTCACCACATCCAAC 3’ |
| *MpPR-1j* | 5` GCCAGTTTGAGCACAGTGACC 3` | 5` AGTTGGAAAAATGTGGGTCGTCA 3` |
| *MpPR-1k* | 5` CGATAGGTCTACAGGGTGATTTC 3` | 5` AGAACTATCCCCGCTTGAATAC 3` |
